# Mesenchymal Tissue-Driven Gene Programs Identify EMP3 as a Key Biomarker of Aggressiveness in Undifferentiated Sarcomas

**DOI:** 10.3390/ijms27073309

**Published:** 2026-04-06

**Authors:** Eun-Young Lee, Ahyoung Cho, Seog Yun Park, June Hyuk Kim, Hyun Guy Kang, Jong Woong Park, Jae Hyang Lim, Joonha Kwon, Hye Jin You

**Affiliations:** 1 Cancer Microenvironment Branch, Division of Cancer Biology, Research Institute, National Cancer Center, Goyang 10408, Republic of Korea; eylee@ncc.re.kr (E.-Y.L.); 76961@ncc.re.kr (A.C.); 2 Department of Microbiology, College of Medicine, Ewha Womans University, Seoul 07804, Republic of Korea; jlim19@ewha.ac.kr; 3 Department of Pathology, National Cancer Center Hospital, National Cancer Center, Goyang 10408, Republic of Korea; 11740@ncc.re.kr; 4 Department of Cancer Biomedical Science, National Cancer Center-Graduate School of Cancer Science and Policy, National Cancer Center, Goyang 10408, Republic of Korea; docjune@ncc.re.kr (J.H.K.); ostumor@ncc.re.kr (H.G.K.); 5 Department of Orthopaedic Surgery, Center for Sarcoma, National Cancer Center Hospital, National Cancer Center, Goyang 10408, Republic of Korea; jwpark82@ncc.re.kr; 6 Rare and Pediatric Cancer Branch, Division of Rare and Refractory Cancer, Research Institute, National Cancer Center, Goyang 10408, Republic of Korea; 7 Medical Engineering Branch, Division of Technology Convergence, Research Institute, National Cancer Center, Goyang 10408, Republic of Korea; 8 Department of Public Health & AI, National Cancer Center-Graduate School of Cancer Science and Policy, National Cancer Center, Goyang 10408, Republic of Korea; joon2k@ncc.re.kr; 9 Surgical Oncology Branch, Division of Clinical Research, Research Institute, National Cancer Center, Goyang 10408, Republic of Korea; 10 Bioinformatics Branch, Division of Cancer Data Science, Research Institute, National Cancer Center, Goyang 10408, Republic of Korea

**Keywords:** spatial transcriptome, undifferentiated pleomorphic sarcoma, biomarker, undifferentiated sarcoma, soft tissue sarcoma, integrated analysis

## Abstract

Undifferentiated sarcomas (USs), including undifferentiated pleomorphic sarcoma (UPS), are aggressive mesenchymal malignancies with limited molecular biomarkers for prognostic assessment and therapeutic stratification. Expression-based markers may provide insight into tumor aggressiveness and clinical outcomes. Here, we performed integrative transcriptomic and spatial analyses to identify differentially expressed genes (DEGs). By comparing normal tissues with sarcoma tumors and sarcoma tumors with cell lines. Intersection and clustering analyses were conducted to define shared expression programs, which revealed a subset of DEGs enriched in epithelial-mesenchymal transition (EMT)-related pathways. CosMx spatial transcriptomics was applied to xenograft tumors derived from two UPS cell lines to resolve tumor-intrinsic signatures. The National Cancer Center Cohort samples were used for validation, and immunohistochemistry confirmed the expression in thirty US tissues. Spatial transcriptomic profiling identified mesenchymal tissue–driven gene expression programs in UPS xenografts. Across bulk RNA-seq and spatial data, epithelial membrane protein 3 (EMP3) consistently emerged as highly expressed in US tissues and cell lines. EMP3 is a robust mesenchymal-associated biomarker linked to EMT, tumor progression, and clinical outcomes in USs, supporting its potential utility as a prognostic indicator and therapeutic target.

## 1. Introduction

Mesenchymal tumors arise in virtually all anatomic sites and are characterized by profound morphological and molecular heterogeneity. Soft tissue sarcomas (STSs) exemplify this diagnostic and biological complexity [1,2,3,4]. In particular, complex karyotype STSs, which lack distinct or pathognomonic molecular alterations, remain challenging to classify and require further elucidation to enable more precise diagnosis and the development of effective therapeutic strategies. In contrast, subtypes characterized by specific chromosomal translocations or gene fusions—often referred to as simple karyotype STSs, such as synovial sarcoma and Ewing sarcoma—are comparatively more straightforward to diagnose [2,5,6,7].

Among complex karyotype STSs, poorly differentiated or unclassified entities—including undifferentiated sarcomas (USs)—remain particularly challenging. According to the fifth edition of the World Health Organization (WHO) Classification of Soft Tissue and Bone Tumors [4], US encompasses undifferentiated pleomorphic sarcoma (UPS), undifferentiated round cell sarcoma (URS), undifferentiated spindle cell sarcoma (USS), and undifferentiated epithelioid sarcoma (UES). Although some tumors initially classified as USs have been reassigned to specific molecular subtypes based on recurrent genetic rearrangements involving genes such as *EWSR1*, *BCOR*, or *NTRK* [4,8], a substantial proportion remain biologically heterogeneous with poorly defined molecular drivers and clinical behavior.

Recent advances in high-throughput and integrative molecular profiling technologies have enhanced our understanding of the molecular [9,10,11] and tumor microenvironmental distinctions [12,13,14] among sarcoma subtypes, facilitating the development of more precise therapeutic strategies [15,16,17,18,19]. However, robust diagnostic and prognostic biomarkers specifically tailored to US remain limited.

Comprehensive genomic approaches, including whole-genome sequencing, contribute to improved molecular classification and prognostic prediction [20,21,22,23,24,25]. In parallel, emerging single-cell and spatial transcriptomic technologies enable increasingly precise characterization of gene-expression networks within their native tissue context [26,27,28,29]. Single-cell transcriptomics permits detailed interrogation of cellular heterogeneity and intercellular interactions, while spatial transcriptomic platforms preserve tissue architecture to resolve cell-to-cell organization in situ. The CosMx Spatial Molecular Imager (NanoString Technologies, Seattle, WA, USA), for example, provides high-resolution, cell-resolved transcriptomic profiling directly within intact tissue specimens [29]. Such spatially resolved analyses may be particularly valuable for US, given its marked heterogeneity and aggressive clinical behavior.

Biomarkers for diagnosis and prognosis have been extensively investigated across multiple cancer types and have contributed to improved therapeutic strategies and clinical outcomes. In sarcomas, the presence of tertiary lymphoid structures (TLS), particularly those enriched with CD19^+^ or CD20^+^ B cells, has been associated with improved responses to immune checkpoint inhibitors in UPS, the predominant subtype of US [13,30]. For prognostic stratification, the 67-gene CINSARC (Complexity Index in SARComas) signature has been proposed as a robust predictor of metastatic risk in STSs [31]. In the original study, US comprised 38% and 37% of cases in the two analyzed cohorts. The prognostic value of this signature has subsequently been validated in multiple independent cohorts and prospective clinical studies [15,16,17,18,19]. However, USs, including UPS, were not the predominant histologic subtypes represented in these validation studies. Notably, the CINSARC genes are primarily associated with cell cycle regulation and proliferation—such as *CCNA2*, *CCNB1*, *CDC2*, and *PLK4*—rather than mesenchymal differentiation or lineage-specific characteristics, underscoring the need for additional biologically informative biomarkers in USs.

Epithelial membrane protein 3 (*EMP3*) is a 163-amino-acid transmembrane protein that was independently identified by two research groups [32,33]. One study demonstrated its biochemical similarity to *EMP1*, *EMP2*, and peripheral myelin protein 22 (*PMP22*), a protein implicated in hereditary neuropathies [33], whereas the other characterized *EMP3* as a tumor-associated membrane protein in c-Myc–driven murine brain tumors [32]. Consistent with its initial identification in a brain tumor model, aberrant *EMP3* expression has been implicated in glioblastoma, pediatric neuroepithelial tumors, and related oncogenic processes [34,35,36,37]. Beyond central nervous system tumors, *EMP3* has been investigated in a variety of epithelial-derived malignancies and related pathological conditions [38], including renal fibrosis [39], thyroid carcinoma [40], oral squamous cell carcinoma [41], gastric cancer [42] and carcinomas of unknown primary, predominantly metastatic carcinomas [43]. However, its role in mesenchymal malignancies, including STS—particularly US subtypes—remains largely unexplored and warrants further investigation.

In this study, we identified differentially expressed genes (DEGs) through transcriptome profiling of US tissues and normal tissues obtained from our previous study [11], and further refined the candidates using a US-driven cell line transcriptome dataset [44]. Moreover, single-cell panel hybridization analysis of xenograft tumor tissues derived from two US cell lines [44], performed using the CosMx platform, enabled the identification of shared cell cluster–associated genes. Based on these integrative cross-analyses, we investigated whether *EMP3* could serve as a potential biomarker for US progression and prognosis.

## 2. Results

### 2.1. Identification of US-Specific Cancer Cell Biomarkers by Transcriptome Profiling

To identify US-specific DEGs, we integrated two transcriptomic datasets obtained from the European Nucleotide Archive (ENA; PRJEB24352) and the Gene Expression Omnibus (GEO; GSE213936) [11,44]: one derived from patient tumor tissues and matched normal tissues, as previously described [11], and another generated from two UPS cell lines (KNCC-STS1 and KNCC-STS2) established in our laboratory [44]. In our previous study, we characterized fourteen STS specimens (US, myxofibrosarcoma, and leiomyosarcoma) with matched normal tissues at the genomic and transcriptomic levels [11]. Tumors were obtained from various anatomical soft tissue sites, predominantly skeletal muscle, with corresponding normal tissues collected from comparable regions. Among the fourteen tumors, six were classified as US (4 UPS, 1 URS, and 1 USS). To identify US-specific biomarker candidates, we first determined DEGs in six US tumor tissues compared with seven matched or comparable normal tissues.

**Figure 1:**
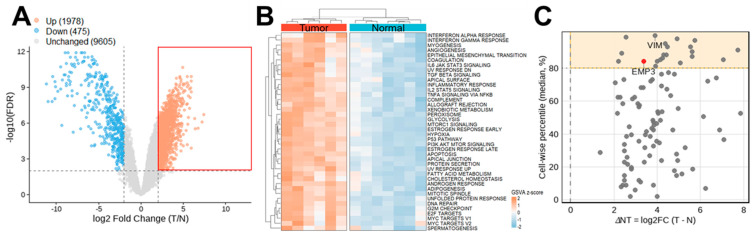
Integrated transcriptomic analysis identifying EMT-associated upregulated DEGs in US tissues and tissue-derived cell lines. (**A**) Volcano plot showing DEGs between US tumors and adjacent normal tissues. A total of 1978 DEGs upregulated in tumors (log_2_ fold change > 2, FDR < 0.01) were selected (red box) for subsequent gene set variation analysis (GSVA). (**B**) Heatmap showing significantly enriched Hallmark pathways in US tumors identified by GSVA (FDR < 0.05). (**C**) Quadrant spotlight plot illustrating the integrated analysis of tumor–normal expression differences (ΔNT) and relative expression ranking in US cell lines.

In total, 12,058 protein-coding genes passed expression-based quality control (QC) among 55,751 quantified genes and were subjected to differential expression analysis, as described in Section 4.2. A total of 2453 DEGs were identified between tumor and adjacent normal tissues using a fold change > 4 and a false discovery rate (FDR) < 0.01 (Figure 1A). The volcano plot, showing log_2_ fold change on the *x*-axis and −log_10_(FDR) on the *y*-axis, illustrates the distribution of these genes. Among them, 1978 were upregulated and 475 were downregulated, whereas 9605 genes did not meet the defined significance thresholds (Figure 1A).

We next analyzed the 1978 upregulated genes to identify pathways associated with US tumor characteristics. The MSigDB Hallmark-based gene set variation analysis (GSVA) revealed multiple pathways significantly enriched in tumors compared with normal tissues (FDR < 0.05) (Figure 1B). Tumors exhibited increased activity of gene programs related to epithelial–mesenchymal transition (EMT), TGF-β signaling, TNFα signaling via NF-κB, IL6–JAK–STAT3 signaling, and inflammatory response. Enhanced enrichment of proliferation-associated Hallmarks—including E2F targets, G2M checkpoint, MYC targets, and mitotic spindle—indicated elevated cell cycle activity concomitant with EMT programs. In addition, metabolic reprogramming was evident, with altered enrichment of glycolysis, fatty acid metabolism, cholesterol homeostasis, and heme metabolism pathways.

We examined the 101 DEGs annotated to the MSigDB Hallmark EMT gene set to identify candidate biomarkers for US tumors (Supplementary Table S1). Transcriptomic data from the KNCC-STS1 and KNCC-STS2 cell clones [44] (GEO; GSE213936) were incorporated to prioritize genes highly expressed in US cell lines. As the datasets were generated independently without shared normalization factors, an integrative analytical approach was applied to enable cross-dataset comparison as described in Section 4.2.3. Integrated analysis identified EMT genes exhibiting both tumor upregulation and high relative expression in KNCC-STS1 and KNCC-STS2 cells. As shown in the quadrant-based scatter plot, genes meeting these criteria were highlighted in a yellow box, resulting in the selection of 21 EMT-associated genes (Figure 1C). These genes were primarily associated with extracellular matrix (ECM) organization and mesenchymal activation. Collagen structural and biosynthetic genes (*COL1A1*, *COL1A2*, *COL6A2*, *SERPINH1*, and *PPIB*) indicated active matrix production, whereas remodeling regulators (*MMP2*, *TIMP1*, and *SERPINE1*) suggested dynamic ECM turnover. Adhesion and cytoskeletal components (*ITGB1*, *CD44*, *FLNA*, *TPM4*, and *VIM*) were consistent with enhanced migratory capacity. EMT-related signaling mediators, including *EMP3*, *TGFBI*, *TNFRSF12A*, and *SERPINE1*, further supported activation of an invasive mesenchymal transcriptional program. Together, these findings define a collagen-producing, matrix-remodeling mesenchymal phenotype in USs.

### 2.2. Spatial Transcriptome-Based Cell Clustering in Tumor Tissues from KNCC-STS1 and KNCC-STS2 Xenograft Models

To further delineate tumor-intrinsic transcriptional programs, spatial transcriptomic profiling was performed on xenograft tumors derived from the UPS cell lines KNCC-STS1 and KNCC-STS2 [44] using the CosMx™ Spatial Molecular Imager (SMI) platform with the 1000-gene Human Universal Cell Characterization RNA Panel.

High-resolution spatial mapping enabled single-cell–level detection of RNA transcripts within preserved tumor architecture. Spatial clustering analysis identified distinct cellular populations, including tumor and stromal components. Because the xenograft models were generated using human US cell lines (KNCC-STS1 and KNCC-STS2), downstream analyses focused specifically on tumor cells. To ensure precise characterization of tumor-derived populations, CD45-expressing immune cells were excluded from further analysis.

**Figure 2:**
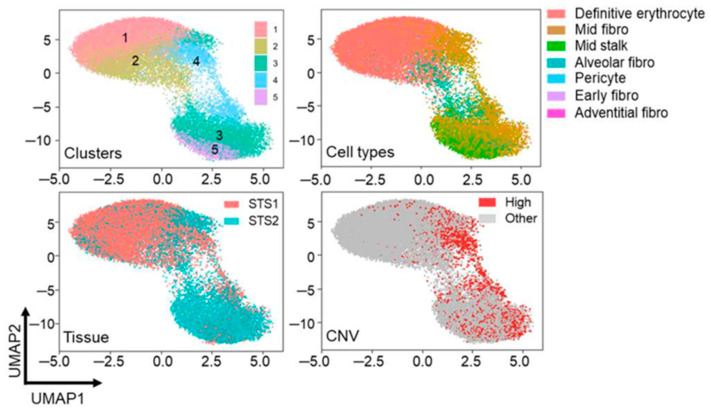
UMAP visualization of Seurat clusters and sample annotations in CosMx spatial transcriptomic data from KNCC-STS1 and KNCC-STS2 xenograft tumors. Uniform Manifold Approximation and Projection (UMAP) of Seurat-derived clusters and associated sample annotations, including cluster identity, tissue origin, cell type, and CNV-high status. Spatial transcriptomic data were processed to identify five clusters (Clusters 1–5; (**top left**)). Clusters were further characterized according to CellTypist-derived fetal_label cell types (**top right**), tissue distribution (**bottom left**), and CNV-high status (**bottom right**). In each UMAP plot, categories are represented by distinct colors as indicated in the corresponding legends.

In total, 32,346 cells from KNCC-STS1 tumors and 16,298 cells from KNCC-STS2 tumors were initially detected by the CosMx Spatial Molecular Imager. After restricting analyses to selected fields of view (FOVs), excluding CD45-positive cells, and applying quality control filtering as described in Section 4.3.2, singlet cells were retained for downstream analysis. This resulted in 15,729 cells from KNCC-STS1 tumors and 9281 cells from KNCC-STS2 tumors with expression profiles across the 1000-gene panel. These cells were subjected to integrated analysis using *Harmony* for batch correction, followed by clustering and Uniform Manifold Approximation and Projection (UMAP) visualization in the *Seurat* package (Figure 2).

Unsupervised clustering identified five distinct tumor cell clusters (Clusters 1–5; Figure 2, top left). Cells from the two tumor tissues were largely separated according to tissue origin (Figure 2, bottom left), although partial overlap was observed. Notably, overlapping regions corresponded to the copy number variation (CNV)-high cell population (Figure 2, bottom right), suggesting shared tumor-intrinsic genomic features across xenograft models.

Because these tumors were derived from mesenchymal US cells rather than epithelial tumors [44], cell type annotation was primarily performed using *CellTypist* with the *fetal_label* module (Figure 2, top right) [45]. This was selected to better capture mesenchymal developmental states and enabled classification of tumor cells into mesenchymal-associated cell populations.

**Figure 3:**
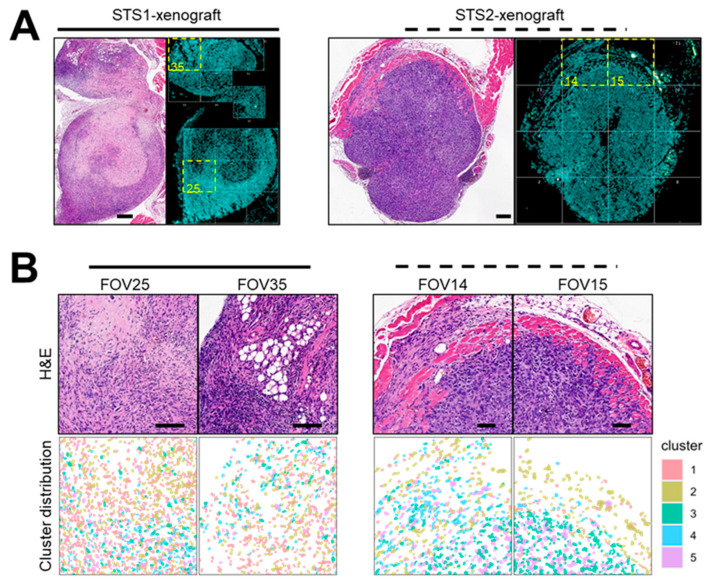
Tumor tissues, cell segmentation maps with FOV annotation, and cluster distribution across FOVs. (**A**) Tumor tissues obtained from xenograft models derived from KNCC-STS1 ((**left**), solid line) and KNCC-STS2 ((**right**), dotted line) cell lines. Cell segmentation maps with FOV annotations are shown adjacent to the corresponding H&E images in each panel. Highlighted FOVs indicate the regions selected for magnified images in (**B**). (**B**) Representative H&E images (**top**) and corresponding cluster distributions (**bottom**) within cell segmentation maps of selected FOVs from KNCC-STS1 ((**left**), solid line) and KNCC-STS2 ((**right**), dotted line) xenograft models. Five clusters are annotated with distinct colors, as indicated in the color index: Cluster 1 (pink), Cluster 2 (mustard), Cluster 3 (green), Cluster 4 (sky blue), and Cluster 5 (violet).

Next, spatial transcriptomic cell clusters were projected onto tissue space using slide-level coordinate information, and CosMx-derived polygon boundaries were reconstructed to generate cell segmentation maps (Figure 3). Cells belonging to each cluster were visualized across FOVs and are shown in distinct colors to illustrate their regional distribution within the tissue (Figure 3B). Spatial remapping of Seurat clusters revealed marked spatial compartmentalization. Notably, Cluster 4 cells (sky blue) displayed a distinct distribution pattern, predominantly localizing to peripheral regions adjacent to surrounding tissues (e.g., skeletal muscle) rather than being uniformly distributed throughout the tumor core. In contrast, Clusters 3 (green) and 5 (violet) were enriched in more central intratumoral zones, consistent with actively growing tumor regions.

### 2.3. Identification of Common Spatial Transcriptome-Driven Biomarkers Across Both Tumor Models

**Figure 4:**
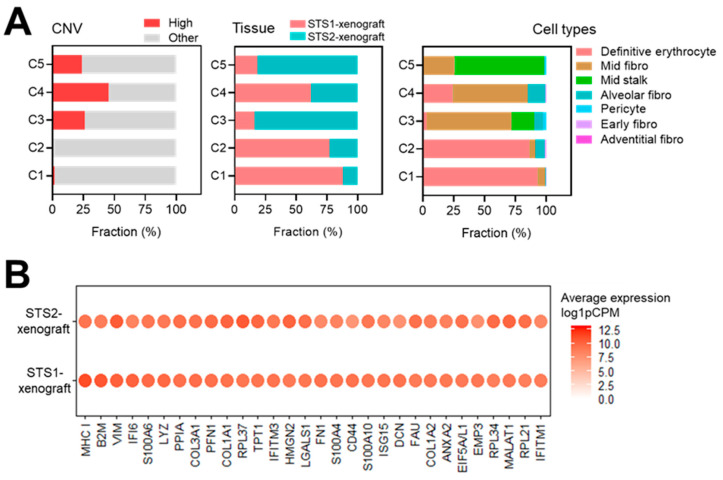
Distribution of CNV-high cells, tissue origin, and cell types across CosMx spatial transcriptome-derived clusters and top 30 genes of Cluster 4. (**A**) Stacked bar plots showing cluster-wise composition according to CNV-high proportion (**left**), tissue origin (**middle**), and cell type (**right**). Colors indicate the annotated categories in each plot. (**B**) Dot plot showing the top 30 genes in CNV-high Cluster 4 (“Mid fibro”) cells grouped by cell types. Dot color intensity is represented by a white-to-red gradient corresponding to gene expression levels shown as normalized counts (log1p-transformed CPM values, log1pCPM).

Profiling of clustered cells according to CNV-high status, tissue of origin, and cell type annotation identified Cluster 4 as a shared population between the two US cell line–derived xenograft tumors (Figure 4A). Within this cluster, a predominant subset annotated as “Mid fibro” and classified as CNV-high was selected for further analysis to identify distinctively expressed genes (Figure 4B).

The top 30 genes from this CNV-high “Mid fibro” subset, derived from both tumor tissues, were ranked according to mean expression levels (log1p-transformed CPM). Notably, several ECM components, including *COL1A2*, *FN1*, *COL1A1*, and *COL3A1*, as well as the mesenchymal marker *VIM* (vimentin), were among the most highly expressed genes. These findings support the validity of our selection strategy and indicate enrichment of mesenchymal-associated gene programs within this cluster.

### 2.4. Integrated Analysis of Bulk RNA-Seq and Spatial Transcriptomics Identifies EMP3 as a Distinct Candidate for US Tumors

**Figure 5:**
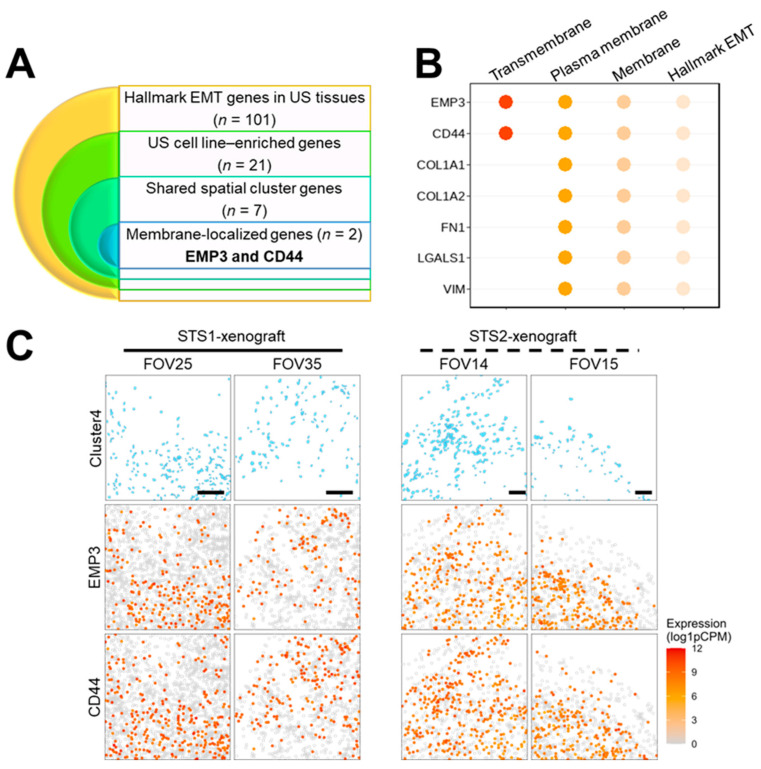
Stepwise selection of EMT-associated candidate genes, membrane topology analysis, and spatial mapping of Cluster 4 in USs. (**A**) Schematic representation of the stepwise filtering strategy. Among 101 upregulated DEGs annotated to the MSigDB Hallmark EMT gene set in US tissues, 21 genes were highly expressed in US cell lines (KNCC-STS1 and KNCC-STS2). Of these, seven genes were shared between two xenograft tumors within a spatial transcriptomic cluster. Two of these genes were further annotated as membrane-localized proteins. (**B**) Dot plot showing the sequential selection of the seven shared DEGs and their membrane topology annotation. (**C**) Spatial maps showing cells classified as Cluster 4 (**top**) and the expression of *EMP3* (**middle**) and *CD44* (**bottom**) across FOVs with cell segmentation. Scale bars, 50 μm. Dot color intensity is represented by a grey-to-red gradient corresponding to gene expression levels shown as normalized counts (log1pCPM).

Next, we performed a cross-sectional integrative analysis to identify biomarker candidates with potential applicability beyond drug targeting, including tumor cell–directed diagnostic or therapeutic strategies. To refine candidate genes, we applied a stepwise intersection approach (Figure 5A,B). Specifically, the top 30 genes enriched in the CNV-high “Mid fibro” subset from both US cell line-derived xenograft tumors were intersected with the EMT-refined, tumor-enriched 21-gene set derived from bulk tumor–normal RNA sequencing and the STS cell line transcriptome panel. Because cell-surface localization is a critical feature for diagnostic and therapeutic targeting, candidate genes were further annotated for membrane association, plasma membrane localization, and transmembrane or integral membrane topology. This annotation-based filtering retained two genes, *EMP3* and *CD44*, both demonstrating consistent membrane-localization evidence (Figure 5B).

These candidates were prioritized because they satisfied three independent criteria: (i) enrichment within the CNV-high tumor compartment identified by spatial transcriptomics, (ii) reproducible upregulation in bulk tumor RNA-seq datasets, and (iii) predicted cell-surface localization compatible with antibody-based detection or targeting strategies. Collectively, integrative analysis of bulk and spatial transcriptomic data converged on two membrane-associated genes as high-confidence candidate sarcoma biomarkers.

The spatial expression patterns of *EMP3* and *CD44* were further examined in relation to Cluster 4 (Figure 5C). Cells expressing *EMP3* or *CD44* predominantly co-localized with Cluster 4 regions in the FOV-based cell segmentation maps. This spatial concordance supports the association of these genes with the mesenchymal-enriched tumor compartment and reinforces their potential as candidate biomarkers for mesenchymal-type sarcoma, with possible applicability in diagnostic stratification and therapeutic targeting.

Notably, one of the two retained candidates was *CD44*, a well-established cell-surface marker associated with tumor-initiating and cancer stem–like cell populations [46]. *CD44*-mediated hyaluronan signaling has been implicated in tumor invasion, metastatic progression, and therapeutic resistance across multiple malignancies [47].

The second candidate, *EMP3*, is a tetraspan membrane protein reported as a prognostic and biologically relevant marker in several cancers, including glioblastoma [34,35,36,39,40,41,42,48,49,50,51,52]. *EMP3* has been widely discussed as a clinically relevant cancer-associated molecule; however, to our knowledge, its role in STS, especially US, has not been previously characterized. Given its membrane localization and the lack of prior investigation in USs, we further evaluated *EMP3* protein expression.

### 2.5. Validation of EMP3 Expression in US and Other Cancer Cell Lines

**Figure 6:**
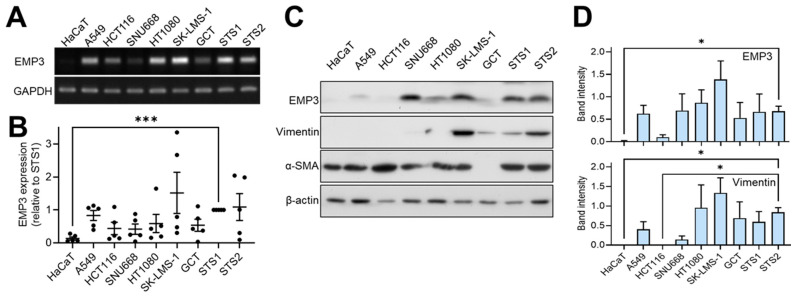
Expression of mesenchymal marker proteins and EMP3 in various cancer cell lines. (**A**,**B**) Cells in the exponential growth phase were harvested and subjected to RT-PCR (**A**) and quantitative real-time PCR (qRT-PCR) (**B**) as a scatter plot. Relative gene expression levels were calculated using the 2^−ΔΔCt^ method with GAPDH as the internal reference gene and normalized to EMP3 expression in KNCC-STS1 cells. Data are presented as mean ± S.E. from at least four independent experiments. (**C**) Protein expression levels of EMP3, Vimentin, and α-SMA were analyzed by immunoblotting. β-actin was used as a loading control. (**D**) Densitometric analysis of immunoblot bands was performed, and protein expression levels were normalized to β-actin. Data are presented as mean ± S.E. from at least four independent experiments (* *p* < 0.05, and *** *p* < 0.001) by GraphPad Prism (version 10.0; GraphPad Software, Boston, MA, USA).

We next examined EMP3 expression across a panel of cancer cell lines. Immortalized human keratinocyte HaCaT cells were included as an epithelial control (Figure 6). At the mRNA level, EMP3 was expressed in most cancer cell lines but was minimally detected in HaCaT cells, as determined by quantitative real-time PCR (qRT-PCR) (Figure 6A,B). Notably, EMP3 expression was significantly higher in KNCC-STS1 cells compared with HaCaT cells. At the protein level, EMP3 and vimentin were predominantly expressed in mesenchymal cancer cell lines, including HT1080, SK-LMS-1, GCT, KNCC-STS1, and KNCC-STS2, whereas their expression was low or absent in HaCaT cells and in epithelial cancer cell lines such as HCT116. In contrast, α-smooth muscle actin (α-SMA) was broadly expressed across cell lines regardless of epithelial origin. Notably, cancer cell lines with EMT characteristics, such as A549 and SNU668, exhibited higher EMP3 expression relative to vimentin (Figure 6C,D). These findings are consistent with enrichment of EMP3 expression in mesenchymal-type cancers, including STS, particularly USs.

### 2.6. Implication of EMP3 in Motility of the US Cancer Cell Line KNCC-STS1

**Figure 7:**
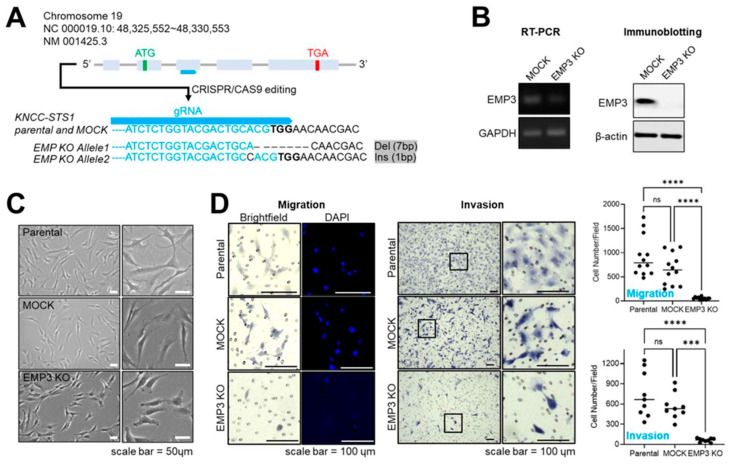
Functional involvement of EMP3 in cellular motility in the US cancer cell line KNCC-STS1. (**A**) Simplified gene structure of *EMP3* for CRISPR-Cas9–mediated genomic editing in KNCC-STS1, a US cancer cell line. Light blue boxes represent exons, gray indicates introns, and blue indicates guide RNA (gRNA). Edited genomic DNA sequences of *EMP3* knockout (KO) cell alleles generated by gRNA are shown. Deleted regions are indicated as “–” with the number of base pairs (bp). (**B**) Expression of EMP3 at the RNA (**left panel**) and protein levels (**right panel**) in MOCK and *EMP3* KO cells established from the KNCC-STS1 cell line. GAPDH or β-actin was used as a loading control. Data shown are representative of at least three independent experiments. (**C**) Cell morphology of KNCC-STS1 parental, MOCK, and *EMP3* KO cells. Scale bars: 50 μm. (**D**) Migration and invasion assays of KNCC-STS1 parental, MOCK, and *EMP3* KO cells. Data shown are representative of at least three independent experiments. Data are presented as mean ± S.E. from at least four independent experiments (*** *p* < 0.001, **** *p* < 0.0001, and ns, non-significant), analyzed using GraphPad Prism (version 10.0; GraphPad Software, Boston, MA, USA).

We next generated an *EMP3* knockout cell line with both alleles edited using the CRISPR-Cas9 system (Figure 7A), resulting in complete loss of EMP3 expression at both the RNA and protein levels (Figure 7B). Morphologically, EMP3 depletion reduced cell length and altered cell shape compared with KNCC-STS1 parental and MOCK cells (Figure 7C). Furthermore, *EMP3* loss significantly reduced cellular motility, as demonstrated by migration and invasion assays using a Transwell system, indicating a critical role of EMP3 in regulating cellular motility.

### 2.7. Spatial Localization and Expression of EMP3 in US Tissues from the NCC STS Cohort

To evaluate the clinical relevance of EMP3, we assessed whether it could serve as a prognostic biomarker candidate in USs. Immunohistochemical analysis was performed on 30 US tissue samples from the NCC STS cohort [53]. The cohort comprised four US subtypes: UPS (*n* = 23), USS (*n* = 5), UES (*n* = 1), and US, not otherwise specified (US, *n* = 1) (Table 1). All specimens were prepared from formalin-fixed, paraffin-embedded (FFPE) blocks and independently reviewed by two sarcoma-specialized pathologists at NCC Hospital according to the standard NCC diagnostic and grading workflow.

For immunohistochemistry evaluation, staining was performed on all 30 US tissues, including negative controls processed without the primary EMP3 antibody. EMP3 expression was assessed using two complementary approaches: (i) semi-quantitative scoring by a sarcoma-specialized pathologist and (ii) digital image analysis using the InForm tissue analysis system, generating two independent scoring metrics (Figure 7).

**Figure 8:**
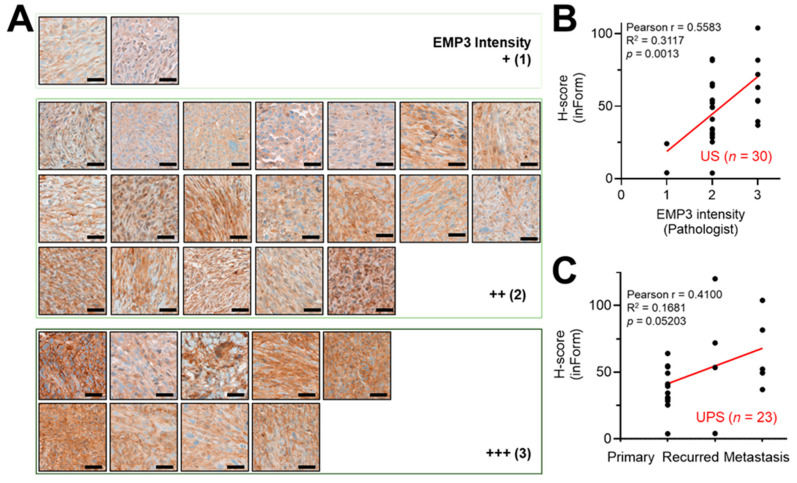
Expression of EMP3 in thirty US tissues from the NCC STS cohort and quantitative histologic analysis. (**A**) Immunohistochemical staining for EMP3 was performed on thirty US tissue specimens from the NCC STS cohort. Representative images of thirty tumor tissues are shown. Scale bars, 50 μm. EMP3 staining intensity was independently evaluated by a board-certified pathologist and scored on a scale from 1 (+) to 3 (+++). Negative controls were processed without primary antibody. (**B**,**C**) Scatter plots showing (**B**) the correlation between H-scores obtained using InForm image analysis software and those evaluated by a pathologist, and (**C**) comparison of H-scores according to tumor status (primary, recurrent, or metastatic). Pearson correlation analysis was performed, and the corresponding R^2^ and *p*-values are indicated. Statistical analyses were conducted using GraphPad Prism (version 10.0; GraphPad Software, Boston, MA, USA).

All US tissues exhibited positive EMP3 expression, ranging from + (1) to +++ (3) according to pathologist-based scoring (Figure 8A). These scores were significantly correlated with H-scores obtained from InForm tissue analyzer–based digital quantification (Figure 8B, top). Notably, H-scores were associated with tumor status, particularly in UPS cases (Figure 8B, bottom). Higher H-scores were observed in advanced, recurrent, and metastatic tumors, whereas lower H-scores were more frequently detected in primary tumor tissues. These findings suggest that elevated EMP3 expression may be associated with disease progression in US, especially in UPS.

The expression pattern of EMP3 was further characterized at both spatial and cellular levels. EMP3 was detected in both membranous and cytoplasmic compartments. Notably, exclusive cytoplasmic localization was observed in approximately 50% of US tissues, suggesting a potential association between subcellular localization and tumor cell status (Figure 8A).

### 2.8. EMP3 as a Prognostic Biomarker Candidate for US Progression and Survival

**Figure 9:**
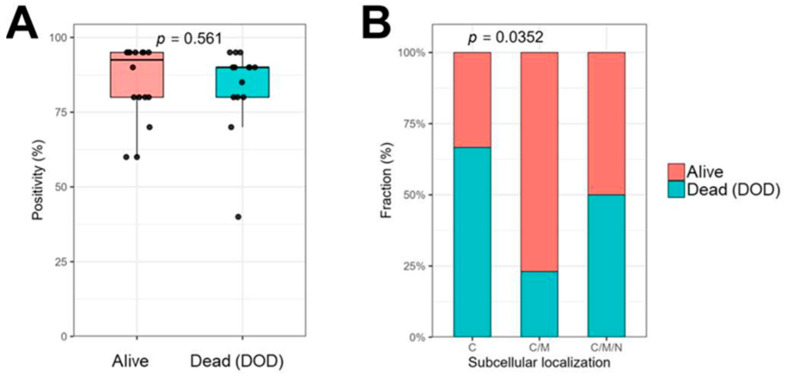
Association of EMP3 expression and subcellular localization with survival in US tissues. Immunohistochemical evaluation of EMP3 expression (**A**) and localization (**B**) was analyzed according to patient survival status (alive vs. died of disease, DOD). (**A**) Box-and-jitter plot showing EMP3 positivity (%) stratified by survival group. Statistical significance was assessed using the Wilcoxon rank-sum test. (**B**) Stacked bar plot showing the distribution of EMP3 subcellular localization between survival groups. Statistical analysis was performed using Fisher’s exact test.

To investigate whether EMP3 expression or subcellular localization was associated with clinical outcomes, we analyzed their relationship with patient survival status. Given the limited cohort size (*n* = 30), patients were stratified into two groups: Alive and Died of Disease (DOD). Differences in EMP3 positivity (%) between groups were assessed using the Wilcoxon rank-sum test (Figure 9A). No statistically significant association was observed. This lack of significance may reflect the small sample size and the absence of time-to-event–based survival analysis in the present cohort.

Interestingly, EMP3 subcellular localization, as determined by pathologist assessment, was significantly associated with patient survival status in the NCC cohort (Fisher’s exact test, Figure 9B). Localization patterns were categorized as cytosolic (C, *n* = 15), cytosolic and membranous (C/M, *n* = 13), or cytosolic, membranous, and nuclear (C/M/N, *n* = 2).

To further investigate the prognostic relevance of EMP3, we analyzed the TCGA Pan-Cancer Atlas sarcoma cohort (Supplementary Table S2). A total of 49 UPS cases with available RNA expression and survival data were included. Cox regression analysis indicated a potential association between EMP3 expression and progression-free survival (PFS); however, due to the limited sample size, these findings should be interpreted cautiously and require validation in larger cohorts.

## 3. Discussion

In this study, we identified DEGs between normal and US tissues using previously published transcriptomic data [11]. These candidate genes were further refined using transcriptomic profiles of two metastatic UPS-derived cell lines [44] and categorized according to their involvement in EMT–related processes, resulting in a 21-gene EMT-enriched signature (Figure 1). Subsequent spatial transcriptomic profiling of xenograft tumors derived from these two cell lines revealed five distinct cellular clusters defined by phenotypic and transcriptomic characteristics using multiple bioinformatic approaches. Notably, one cluster comprised comparable cellular populations across both tumor models while exhibiting distinct DEG enrichment patterns (Figure 2, Figure 3 and Figure 4). By integrating bulk and spatial transcriptomic analyses, we identified overlapping candidate genes and prioritized those encoding membrane-localized proteins for further validation and potential translational application (Figure 5). Among these, EMP3, CD44, and VIM emerged as key candidates. We further focused on EMP3 as a novel biomarker candidate for US and evaluated its expression by immunohistochemistry in US tumor tissues, as well as across epithelial- and mesenchymal-origin cancer cell lines, to validate its spatial and cellular expression patterns (Figure 6, Figure 7 and Figure 8). Finally, we assessed its association with clinicopathologic features and clinical outcomes (Figure 9).

EMT is a developmental program that occurs during embryogenesis and plays a critical role in tissue morphogenesis and organ formation [54,55]. In cancers arising from epithelial tissues, loss of epithelial integrity and acquisition of mesenchymal features are considered analogous to developmental EMT [54,56,57,58]; however, the molecular programs underlying cancer-associated EMT continue to be refined [59,60]. More recently, EMT in cancer has been recognized as a spectrum of transitional states rather than a binary process, leading to the concepts of epithelial–mesenchymal plasticity (EMP) or partial EMT [61,62]. Accordingly, the molecular networks governing EMT-related gene programs warrant further clarification across diverse tumor types, including both epithelial- and mesenchymal-origin malignancies.

Markers such as vimentin and α-smooth muscle actin (α-SMA) have traditionally been used to validate mesenchymal characteristics in epithelial-derived tumors undergoing EMT. However, in STSs, particularly US subtypes, these markers do not necessarily reflect malignant transformation, as these tumors are inherently of mesenchymal origin. Although emerging studies suggest functional roles for vimentin in cancer progression, invasion, and cellular plasticity [63,64], much of this evidence is derived from epithelial cancers undergoing EMT rather than from primary mesenchymal malignancies. Therefore, the biological significance of classical EMT markers in STSs remains to be fully elucidated.

Here, we applied the CosMx Universal Cell Characterization RNA Panel for spatial transcriptomic profiling to delineate the cellular architecture of US tumor tissues, emphasizing intrinsic cellular states and spatial organization rather than relying solely on predefined cancer-specific markers. This approach enabled the identification of tumor-intrinsic transcriptional programs associated with mesenchymal activation and EMT-related plasticity within the spatial context of the tumor microenvironment in US.

Importantly, our integrative analysis of bulk and spatial transcriptomic data revealed membrane-associated genes enriched in CNV-high tumor compartments, highlighting potential functional drivers of mesenchymal tumor progression. These findings extend current understanding of mesenchymal tumor biology by linking spatially defined cellular states with EMT-related gene programs in US. Nevertheless, further mechanistic studies are warranted to clarify the regulatory networks and functional contributions of these candidate genes in sarcoma development and progression.

*EMP3* was initially identified in neuropathy models and brain tumor tissues [32,33] and has since been implicated in a variety of malignancies, predominantly those of epithelial origin [34,35,36,40,41,42,49,50,51,52]. As a membrane protein, EMP3 has been reported to interact with *CD44* [48] in erythrocyte for antigen presentation and the P2X7 receptor [65] in HEK293 cell models, where it has been associated with membrane blebbing and apoptotic processes. In addition, several studies have suggested a role for EMP3 in regulating receptor tyrosine kinase stability and downstream signaling pathways [38]. Despite these observations, the precise molecular functions of *EMP3* and its integration within broader protein interaction networks remain incompletely understood. Furthermore, the subcellular localization patterns of EMP3 in US tissues, together with its association with patient survival status (Figure 7 and Figure 8), prompted us to investigate the relationship between EMP3 expression and cellular localization in both epithelial and mesenchymal cancer cells. The potential roles of EMP3 in diverse cellular processes are currently under investigation.

Interestingly, EMP3 has been reported to associate with leucyl-tRNA synthetase (LARS) in breast cancer, linking EMP3 to metabolic regulation and cellular transformation [52]. This interaction suggests a potential connection between amino acid sensing, protein synthesis, and tumor cell proliferation. The relationship between amino acid metabolism and malignant transformation is increasingly recognized as a critical component of cancer biology. However, in our transcriptomic analyses of US tissues and derived cell lines, LARS expression was neither significantly altered nor correlated with EMP3 expression. These findings suggest that EMP3-associated regulatory mechanisms in US may differ from those reported in epithelial cancers such as breast carcinoma.

In mesenchymal tissues, the molecular drivers underlying malignant transformation remain incompletely defined. Several studies have suggested roles for CD44 and related molecules in STS biology [46,47], and CD44 has been proposed as a potential marker for monitoring disease progression, including through circulating tumor cells. However, CD44 is a lineage-associated marker expressed in multiple alternatively spliced isoforms, and its specific contribution to STS progression remains unclear. A previous study demonstrated an association between CD44 and EMP3 in erythroid cells in the context of the rare *MAM* blood group [48,51]. Although these findings were observed in a distinct cellular context, they raise the possibility that CD44 and EMP3 may functionally cooperate during sarcoma progression.

In the present study, EMT-associated genes and collagen biosynthetic pathways were predominantly localized within tumor-cell regions rather than stromal compartments, indicating that these programs are intrinsic to malignant cells. EMP3 was strongly enriched in tumor-cell clusters and spatially co-localized with key EMT and ECM remodeling genes, supporting its association with mesenchymal activation in US. We successfully generated an *EMP3* KO cell line with biallelic disruption. The *EMP3* KO cells exhibited distinct morphological changes compared with KNCC-STS1 parental and MOCK cells, along with a marked reduction in cellular motility. These *EMP3* KO cells provide a useful model to investigate the underlying mechanisms by which EMP3 regulates cell motility, thereby contributing to our understanding of US development and progression and suggesting a potential role for EMP3 in tumor progression and metastatic potential. EMP3 was consistently expressed across US tissues, and its subcellular localization was significantly associated with patient survival status. In addition, a higher proportion of EMP3-positive cells was observed in metastatic cases. A significant association was observed between EMP3 RNA expression and survival outcomes, particularly PFS, in the TCGA Pan-Cancer sarcoma cohort (UPS subtype, *n* = 49), after adjusting for age, sex, and ICD-10 classification (Supplementary Table S2). Although validation in larger cohorts is required, these findings suggest that EMP3 may serve as a candidate marker for mesenchymal tumor identification and prognostic stratification. Given its membrane localization, EMP3 may also represent a potential target for diagnostic or therapeutic development.

Overall, spatial transcriptomic analysis demonstrates that the identified mesenchymal gene signature reflects tumor cell–specific biology rather than stromal contamination, underscoring the translational relevance of EMP3 in US.

## 4. Materials and Methods

### 4.1. Ethics Statement

This study was approved by the Ethics Review Board of the National Cancer Center, Korea (IRB No.: NCC2017–0062, approved on 3 August 2017). Written informed consent was obtained from all patients before tissue acquisition, and all samples were stored according to the principles of the Declaration of Helsinki.

### 4.2. Bioinformatical Analysis—Differentially Expressed Genes (DEGs) Selection

#### 4.2.1. Differential Gene Expression Analysis

For DEG selection in undifferentiated sarcoma (US), transcriptomic data from six US tissues and seven matched or anatomically comparable normal tissues from the NCC cohort were analyzed, as previously [11,44]. Genes were retained if they exhibited FPKM ≥ 1 in at least 20% of all samples or in at least 20% of samples within either the tumor or normal group (minimum group size ≥ 2). Of the 55,751 quantified genes, 15,109 passed expression-based quality control criteria. For biological interpretation, analyses were restricted to protein-coding genes annotated in Ensembl GRCh38, excluding pseudogenes and non-coding transcripts, resulting in 12,058 coding genes for downstream analysis. Expression values were log_2_-transformed (log_2_[FPKM + 1]) prior to statistical testing. Differential expression between tumor and normal tissues was assessed using linear models implemented in the *limma* package with empirical Bayes moderation. *p*-values were adjusted for multiple comparisons using the Benjamini–Hochberg method [66,67]. Genes with an adjusted FDR < 0.01 and an absolute log_2_ fold change ≥ 2 were defined as DEGs, yielding 2453 DEGs.

Volcano plots were generated using the ggplot2 package, displaying log_2_ fold change on the *x*-axis and −log_10_(FDR) on the *y*-axis. Genes were classified as upregulated, downregulated, or unchanged according to the thresholds described above. Volcano plots were generated using *ggplot2*, displaying log_2_ fold change on the *x*-axis and −log_10_(FDR) on the *y*-axis. Genes were classified as upregulated, downregulated, or unchanged based on the differential expression thresholds described above.

#### 4.2.2. GSVA

GSVA was performed using MSigDB Hallmark gene sets obtained via the *msigdbr* package. To harmonize pathway scoring with the tumor-upregulated signature used for differential expression analysis, each gene set was restricted to genes classified as tumor-upregulated; gene sets retaining fewer than 10 genes present in the expression matrix were excluded. GSVA scores were computed from log_2_(FPKM + 1) expression values using a Gaussian kernel.

Tumor–normal differences in GSVA scores were assessed using *limma* with empirical Bayes moderation and Benjamini–Hochberg correction [66]. Pathways with an FDR < 0.05 were considered statistically significant. For visualization only, GSVA scores of significant pathways were standardized by row-wise z-score transformation and displayed as hierarchically clustered heatmaps.

#### 4.2.3. Quadrant Spotlight Analysis

To integrate and compare genes across two datasets lacking common normalization factors, the following analytical strategy was applied. For the tissue transcriptome, expression values (FPKM) were transformed as log_2_(FPKM + 1), and tumor–normal differences were quantified as ΔNT = Tumor_median_ − Normal_median_ based on the log_2_-transformed values. For the KNCC-STS1 and KNCC-STS2 transcriptomes (GSE213936) [44], log_2_(FPKM + 1) values were converted to within-sample empirical cumulative distribution function (ECDF)–based percentiles calculated across the 101 (DEGs, Supplementary Table S1). Each gene was then summarized by its median percentile across samples.

The two metrics were merged by gene symbol (dots, *n* = 101) and visualized as a scatter plot, with ΔNT (difference in log_2_[FPKM + 1] medians; tumor minus normal) on the *x*-axis and the median 101-DEG percentile on the *y*-axis. A quadrant-based scatter plot highlighted genes meeting these criteria (log_2_FC > 0; cell_pct_median ≥ 80th percentile within DEGs), resulting in the selection of 21 genes.

#### 4.2.4. Membrane-Protein Annotation and Surface Localization Evidence

Membrane-related annotation evidence for the identified DEGs was retrieved from UniProt and Gene Ontology (GO) cellular component databases. Three binary indicators were assigned to each gene: (i) membrane association, (ii) plasma membrane localization, and (iii) transmembrane or integral membrane topology. Genes were ranked using a weighted evidence scoring system based on these three categories (membrane, plasma membrane, and transmembrane annotations) to prioritize candidates with potential surface localization and to enable visualization of membrane-related annotation strength.

### 4.3. Spatial Transcriptomic Profiling Using CosMx

#### 4.3.1. Sample Preparation for CosMx Spatial Transcriptomic Experiments

Formalin-fixed paraffin-embedded (FFPE) xenograft tumor specimens were generated by subcutaneous injection of human UPS-derived cell lines (KNCC-STS1 and KNCC-STS2) into immunodeficient mice, as previously reported [44]. FFPE tumor blocks were sectioned at 5 μm thickness and mounted onto appropriate slides for spatial transcriptomic analysis. Tissue sections were processed and profiled using the CosMx™ Human Universal Cell Characterization RNA Panel (1000-plex) on the CosMx Spatial Molecular Imager (NanoString Technologies, Seattle, WA, USA). Spatial transcriptomic experiments, including tissue processing, hybridization, imaging, and primary data generation, were performed by Macrogen Inc. (Seoul, Republic of Korea) according to the manufacturer’s protocols.

#### 4.3.2. Data Processing and Quality Control of CosMx Spatial Transcriptomic Data

Spatial transcriptomic data were analyzed in *R* (version 4.4.1; R Foundation for Statistical Computing, Vienna, Austria) using the *Seurat* package. Raw transcript counts were generated using the CosMx Spatial Molecular Imager analysis pipeline (NanoString Technologies). Cell segmentation was performed according to the default CosMx workflow based on nuclear (DAPI, blue channel) and membrane markers (PanCK, red channel; CD45, green channel). Analyses were restricted to human tumor cells from selected fields of view (FOVs). To exclude immune cell populations in xenograft tissues profiled with the CosMx™ Human Universal Cell Characterization RNA Panel (1000-plex), immune cells were identified using imaging-derived CD45 and CD68 intensity features. Only CD45-negative cells were retained for downstream analyses. Quality control filtering was applied to remove low-quality cells based on transcript detection thresholds. Cells with fewer than 20 detected genes (nFeature_RNA < 20) were excluded. Gene expression values were normalized to counts per million (CPM) and log1p-transformed (log1p CPM) for comparative analyses.

Highly variable genes of the panel (*n* = 1000) were selected for downstream dimensionality reduction. Putative doublets were identified using the *scDblFinder* package following conversion to a *SingleCellExperiment* object, and only singlet cells were retained for further analysis.

#### 4.3.3. Characterization of Clusters by Cell Type, Tissue Origin, and Copy Number Variation (CNV)

Normalization was performed using trimmed mean of M-values (TMM) normalization implemented in the *edgeR* package. Normalized data were scaled and subjected to principal component analysis (PCA) in *Seurat*. Batch effects were corrected using the *Harmony* algorithm with tissue_ID specified as the grouping variable. Uniform manifold approximation and projection (UMAP) was generated from the batch-corrected embeddings for visualization.

Cell Type Annotation: Cell type annotation was performed in Python (Python 3.12.7, Python Software Foundation, Wilmington, DE, USA) using CellTypist [45,68]. Predicted labels were imported into *Seurat* as metadata (fetal_label) for downstream analyses. For UMAP visualization, annotation categories represented by fewer than 10 cells were excluded to minimize over-interpretation of rare populations. Cells assigned to these low-frequency labels were omitted only from visualization and compositional summaries but retained in the full dataset for all downstream analyses.

Copy Number Variation (CNV) Inference and Definition of CNV-High Cells: CNV was inferred using *InferCNV* with a two-pass self-referencing strategy applied separately for each tissue_ID. In the first pass, CNV was estimated without predefined reference cells to compute per-cell CNV deviation scores. Reference-like cells were then adaptively selected as the lowest-deviation fraction (~20–40% per tissue), excluding mitotic-high cells, and used as internal references for a second InferCNV run. CNV burden was quantified per cell using two metrics: (i) CNV_level, defined as the median inferred copy number, and (ii) CNV_score, defined as the upper-tail deviation from copy neutrality. CNV scores were optionally smoothed using distance-weighted k-nearest neighbor propagation. *CellTypist*-derived annotations (fetal_label and breast_label) were not used for CNV inference or reference selection and were applied solely for downstream visualization and compositional analyses. CNV-high cells were defined in a tissue-specific manner as those with CNV_score exceeding the tissue-wise mean plus one standard deviation; all remaining cells were classified as CNV-other.

#### 4.3.4. Spatial Visualization of Cluster Topology and Marker Gene Expression in CosMx Tissue Space

Spatial cluster maps were generated by projecting *Seurat*-derived cluster identities onto the CosMx tissue coordinate system using slide-level spatial coordinates (x_slide_mm, y_slide_mm). Cell segmentation boundaries were reconstructed from CosMx polygon vertex coordinates, and individual cells were rendered as polygonal outlines in tissue space.

For each tissue_ID, whole-slide cluster topology was visualized. FOV boundaries were defined based on the spatial extent of segmented cells within each FOV and overlaid as dashed rectangles with corresponding FOV labels.

Spatial expression patterns of EMP3 and CD44 were visualized in tissue space using precomputed log1pCPM. Gene expression levels were overlaid onto segmented cell polygons or centroid coordinates derived from CosMx segmentation data. A fixed absolute color scale was applied across tissues to enable direct comparison of expression intensity between samples. All spatial visualizations were generated using the *ggplot2* package in *R*.

#### 4.3.5. Gene Ranking Within the Cluster 4 Subset

Within the subset of CNV-high cells annotated with the fetal_label “Mid fibro” in Cluster 4, the mean log1p-transformed CPM (log1pCPM) expression value was calculated for each gene across all cells. Genes were ranked according to their mean expression levels, and the top 30 genes were selected for visualization. Tissue-specific expression patterns were displayed using *Seurat*’s *DotPlot* function with a fixed color scale (0–13) to ensure comparability across tissues.

### 4.4. Cell Culture

The human keratinocyte cell line HaCaT, lung carcinoma cell line A549, colon carcinoma cell line HCT116, fibrosarcoma cell line HT1080, leiomyosarcoma cell line SK-LMS-1, and STS cell line GCT were obtained from the American Type Culture Collection (ATCC, Manassas, VA, USA). The gastric cancer cell line SNU668 was obtained from the Korean Cell Line Bank (00668, KCLB, Seoul, Republic of Korea). The human STS cell lines representing undifferentiated pleomorphic sarcoma (UPS), KNCC-STS1 and KNCC-STS2, were established in our previous study [44]. All cell lines were authenticated by short tandem repeat (STR) polymerase chain reaction (PCR) analysis between 2023 and 2025.

HaCaT, A549, HCT116, KNCC-STS1, and KNCC-STS2 cells were cultured in high-glucose Dulbecco’s Modified Eagle Medium (DMEM; Gibco, Thermo Fisher Scientific, Waltham, MA, USA). SNU668 cells were maintained in RPMI-1640 medium (Gibco). GCT cells were cultured in McCoy’s 5A medium (Gibco), whereas HT1080 and SK-LMS-1 cells were maintained in Eagle’s Minimum Essential Medium (EMEM; Corning, NY, USA). All culture media were supplemented with 10% heat-inactivated fetal bovine serum (FBS) and 1× antibiotic–antimycotic solution. Cells were maintained at 37 °C in a humidified incubator with 5% CO_2_ atmosphere.

### 4.5. Generation of EMP3 Knockout (KO) Cell Lines

The clustered regularly interspaced short palindromic repeats (CRISPR)/CRISPR-associated protein 9 (Cas9) system was employed to generate EMP3 knockout (KO) cells using the pSpCas9(BB)-2A-green fluorescent protein (GFP) vector (px458) as previously described [69,70,71]. Guide RNAs (gRNAs) targeting EMP3 were designed using the CHOPCHOP web tool (http://chopchop.cbu.uib.no, 27 June 2023) and cloned into the pSpCas9(BB)-2A-GFP vector, followed by sequence verification. Cells (5 × 10^5^) were seeded and cultured for 24 h prior to transfection with the CRISPR/Cas9 constructs targeting EMP3 or the empty CRISPR/Cas9 vector (MOCK). After 48 h, GFP-positive cells were sorted by fluorescence-activated cell sorting (FACS) and seeded into 96-well plates to allow clonal expansion. Individual colonies were screened by immunoblotting to confirm EMP3 knockout. Two EMP3 KO clones and one MOCK clone were established, and one EMP3 KO clone and one MOCK control were used for subsequent experiments.

### 4.6. RNA Extraction, Reverse Transcription (RT) and Related PCRs

#### 4.6.1. RNA Extraction

Total RNA was extracted using the RNeasy Mini Kit (Qiagen, Hilden, Germany) according to the manufacturer’s instructions. Briefly, at least 1 × 10^6^ cells were lysed in Buffer RLT and homogenized by passing the lysate through a QIAshredder column (Qiagen) to remove cellular debris. The cleared lysates were mixed with an equal volume of 70% ethanol and transferred to RNeasy spin columns for RNA purification. After sequential washing steps, total RNA was eluted in RNase-free distilled water.

RNA concentration and purity were assessed using a NanoDrop 2000 spectrophotometer (Thermo Fisher Scientific). RNA purity was evaluated based on the A260/A280 and A260/A230 absorbance ratios.

#### 4.6.2. RT-PCR and Quantitative(q) RT-PCR

Total RNA (5 µg) was reverse-transcribed into first-strand cDNA using oligo(dT) primers and SuperScript™ III Reverse Transcriptase (Thermo Fisher Scientific) according to the manufacturer’s instructions. cDNA synthesis was performed at 50 °C for 60 min in a total reaction volume of 50 µL containing 10 mM Tris-HCl (pH 8.3), 50 mM KCl, 5 mM MgCl_2_, and 1 mM dNTPs.

For RT-PCR, hot-start PCR was carried out using TOP DNA polymerase (Bioneer, Daejeon, Republic of Korea) with primers designed to amplify specific target genes (Supplementary Table S3), according to previously optimized PCR conditions [72]. PCR products were separated by electrophoresis on 2% (*w*/*v*) agarose gels, stained with ethidium bromide, and visualized using a GelDoc imaging system (Azure 200; Azure Biosystems, Dublin, CA, USA). Band intensities were quantified using ImageJ software (v1.53; National Institutes of Health, Bethesda, MD, USA).

#### 4.6.3. Quantitative Real-Time-PCR (qRT-PCR)

qRT-PCR was performed using FastStart SYBR Green Master (Roche Diagnostics, Mannheim, Germany) on a LightCycler^®^ 96 real-time PCR system (Roche Diagnostics, Basel, Switzerland). For each reaction, 20 ng of cDNA was used as template. Specifically, 1 µL of cDNA was used for GAPDH amplification and 3 µL for EMP3 amplification, together with gene-specific primers. PCR amplification was carried out with an initial denaturation step at 95 °C, followed by 40 cycles of denaturation, annealing, and extension according to the manufacturer’s recommended conditions. Relative gene expression levels were calculated using the 2^−ΔCt^ method, with GAPDH serving as the internal reference gene [73], and graphically presented using GraphPad Prism (version 10.0; GraphPad Software, Boston, MA, USA).

### 4.7. Immunoblotting

Protein samples were separated by sodium dodecyl sulfate–polyacrylamide gel electrophoresis (SDS–PAGE) on 8–15% gels, depending on the molecular weight of the target proteins, and transferred onto polyvinylidene difluoride membranes using a Mini-PROTEAN^®^ Tetra Cell and PowerPac™ Basic system (Bio-Rad, Hercules, CA, USA) at 100 V for 1 h. Membranes were blocked with 5% non-fat dried milk in Tris-buffered saline containing 0.1% Tween-20 (TBST) and incubated overnight at 4 °C with primary antibodies diluted in TBST containing 2% non-fat dried milk (Supplementary Table S4). After three washes with TBST (7 min each), membranes were incubated with horseradish peroxidase (HRP)-conjugated secondary antibodies. Protein bands were detected using the Miracle-Star™ Western Blot Detection System (INtRON Biotechnology, Seongnam, Republic of Korea) and visualized on Amersham™ Hyperfilm™ ECL (Cytiva, Marlborough, MA, USA). β-actin was used as a loading control. Band intensities were quantified using ImageJ software (v1.53; National Institutes of Health, Bethesda, MD, USA) and graphically presented using GraphPad Prism (version 10.0; GraphPad Software).

### 4.8. Migration and Invasion Assay

For the migration assay, cells (1 × 10^4^) were suspended in DMEM and seeded into the upper chamber of an 8-μm pore size Transwell insert (6.5 mm diameter; Corning Costar Corp., Corning, NY, USA). The lower chamber was filled with DMEM supplemented with 10% FBS. After 6 h, the inserts were washed with phosphate-buffered saline (PBS), and migrated cells on the membrane were fixed with 4% formaldehyde and stained with hematoxylin and eosin (H&E; Sigma-Aldrich, Merck, Darmstadt, Germany). For the invasion assay, the upper chamber of the Transwell insert (8-μm pore size) was precoated with Matrigel (1 mg/mL; Corning) prior to seeding. Cells (1 × 10^4^) suspended in DMEM were added to the upper chamber, while the lower chamber contained DMEM supplemented with 10% FBS. After 24 h, the inserts were washed with PBS, and invading cells were fixed and stained as described above.

Each assay was performed at least three times. For quantification, three randomly selected fields per membrane were imaged at 10× magnification using a MATEO FL microscope (Leica Microsystems, Wetzlar, Germany).

### 4.9. Patient Data and Tissue Specimen

Tissue specimens were obtained from US patients by wide resection. Thirty specimens were collected directly during surgery and were properly prepared for subsequent analyses, including histological evaluation. All specimens were centrally reviewed to ensure consistent and accurate subtype classification according to the standard NCC diagnostic workflow by expert sarcoma pathologists at the NCC Hospital, incorporating clinical information, morphologic assessment, and molecular analyses. USs were not defined by specific cytogenetic breakpoints; instead, a histopathology-based classification was applied. To do this, tissue specimens were fixed overnight in 10% (*v*/*v*) neutral-buffered formalin and processed into FFPE tissue blocks. Table 1 lists the clinicopathologic features of the patients and tumors.

### 4.10. Histological Analysis

From FFPE tissue blocks, sections of 4-μm thickness were prepared. Tissue slides were stained with hematoxylin and eosin (H&E) to assess tumor cell density and were reviewed by two pathologists specializing in sarcomas for tumor cell entity, necrosis and so on. Selected slides were subjected to immunohistochemistry using antibodies against Ki-67 (a proliferation marker; ab15580, Abcam, Cambridge, UK) and EMP3 (sc-81797, Santa Cruz Biotechnology Inc., Dallas, TX, USA). Immunoreactivity was visualized using 3,3′-diaminobenzidine (DAB), with hematoxylin used for nuclear counterstaining, at the Laboratory Animal Research Core (NCC facility). All stained slides were independently reviewed and scored by one pathologist specializing in sarcomas.

The stained slides were scanned at 20 × magnification using the Vectra^®^ Polaris™ automated quantitative pathology imaging system (Akoya Biosciences Inc., Lexington, MA, USA), confirmed using Phenochart Viewer version 2.0.1 (Akoya Biosciences Inc.), and quantitatively analyzed using inForm^®^ Tissue Finder software (version 2.6.0, Akoya Biosciences Inc.). For EMP3 expression analysis, representative regions of interest (ROIs) were selected from multiple tissue sections, with negative controls used to define intensity thresholds and scoring (H-Score). Spectral libraries were generated and applied for spectral unmixing, enabling classification of tumor, non-tumor, background, and necrotic regions (Supplementary Figure S1). Image analysis was performed using predefined algorithms that included image preparation, tissue and cell segmentation, object detection, and quantitative scoring. Cells exhibiting a predominant membranous staining pattern were defined as positive, and the staining intensity of each cell was automatically quantified by the software. Threshold and coordinated parameters were applied according to the specific analysis settings, and all tissue images were analyzed using the same workflow to ensure consistency according to the manufacturer’s instruction.

The H-score (histochemical score) is a quantitative IHC interpretation method ranging from 0 to 300, calculated by multiplying the staining intensity (0–3) by the percentage of positive cells (0–100%), resulting in a dynamic range for assessing biomarker expression (stronger, more extensive, and more positive expression).

### 4.11. Statistical Analysis

Pathologist-assessed EMP3 positivity (%) was compared between survival groups (Alive vs. Died of Disease [DOD]) [53] using the Wilcoxon rank-sum test and visualized with box-and-jitter plots. Differences in EMP3 subcellular localization between survival groups were evaluated using Fisher’s exact test and presented as stacked bar plots.

Data are presented as mean ± standard error (S.E.), unless otherwise specified. Comparisons between two groups were performed using Student’s *t*-test. For multiple testing, *p*-values were adjusted using the Benjamini–Hochberg FDR correction. A two-sided *p*-value < 0.05 was considered statistically significant [66,67,74]. All statistical analyses were performed using *R* (version 4.4.1; R Foundation for Statistical Computing, Vienna, Austria) and GraphPad Prism (version 10.0; GraphPad Software, Boston, MA, USA).

## 5. Conclusions

This study identifies EMP3 as a mesenchymal-associated biomarker supported by integrated transcriptomic, spatial, and histopathologic evidence. Elevated EMP3 expression and distinct subcellular localization were associated with aggressive clinicopathologic features, including metastatic disease, in US. These findings position EMP3 as a promising candidate for tumor stratification and prognostic assessment in US, with potential translational implications for diagnostic and therapeutic applications.

## Figures and Tables

**Table 1:** Summary of clinical and pathologic information.

Total Number of Individual	30
Age (years)	65.0 ± 2.3 (43–89)
Sex	
Female	10 (33.3%)
Male	20 (66.7%)
FNCLCC Grade	
Grade I	0 (0.0%)
Grade II	4 (13.3%)
Grade III	26 (86.7%)
Presentation	
Primary	18 (60.0%)
Local recurrence	7 (23.3%)
Metastasis	5 (16.7%)
Subtype	
UPS *	23 (76.7%)
USS	5 (17.9%)
UES	1 (3.3%)
US	1 (3.3%)

Values are presented as number (%) or mean ± SE (range); US, undifferentiated sarcoma; UPS, undifferentiated pleomorphic sarcoma; USS, undifferentiated spindle cell sarcoma; URS, undifferentiated round cell sarcoma; UES, undifferentiated epithelioid sarcoma; FNCLCC, Fédération Nationale des Centres de Lutte Contre Le Cancer. * One patient with primary UPS of bone was included.

## Data Availability

The datasets generated and analyzed during the current study are available in the European Nucleotide Archive (ENA) under accession number PRJEB24352 and in the Gene Expression Omnibus (GEO) under accession numbers GSE213936 and GSE320395, corresponding to patient-derived transcriptomes, US cell line transcriptomes, and CosMx-based spatial transcriptomic data from US xenograft models, respectively.

## Supplementary Materials

The following supporting information can be downloaded at: https://www.mdpi.com/article/10.3390/ijms27073309/s1.

## Abbreviations

The following abbreviations are used in this manuscript:

CNV	Copy number variation
CPM	Counts per million mapped reads
DEG	Differentially expressed gene
DOD	Died of disease
EMT	Epithelial to mesenchymal transition
FDR	False discovery rate
FPKM	Fragment per kilobase of transcript per million mapped reads
GSVA	Gene set variation analysis
STS	Soft tissue sarcoma
US	Undifferentiated sarcoma
UPS	Undifferentiated pleomorphic sarcoma
URS	Undifferentiated round cell sarcoma
USS	Undifferentiated spindle cell sarcoma
