# Supplementary material for: Mesenchymal Tissue-Driven Gene Programs Identify EMP3 as a Key Biomarker of Aggressiveness in Undifferentiated Sarcomas
Source: Int J Mol Sci. 2026 Apr 6;27(7):3309. doi: 10.3390/ijms27073309 (PMC13073760; doi:10.3390/ijms27073309)
Supplement: Supplementary file 1 [file ijms-27-03309-s001.zip › ijms-4201824-supplementary.pdf]

Mesenchymal Tissue–Driven Gene Programs Identify EMP3 as a Key Biomarker of Aggressiveness in Undifferentiated Sarco-mas

Eun-Young Lee[1,†], Ahyoung Cho[1,2,†], Seog Yun Park[3], June Hyuk Kim[4,5,6], Hyun Guy Kang[4,5,7], Jong Woong Park[5,8,9], Jae Hyang Lim[2], Joonha Kwon[8,10] and Hye Jin You[1,4,*]

[1]Cancer Microenvironment Branch, Division of Cancer Biology, Research Institute, National Cancer Center, Goyang, 10408, Republic of Korea; [2]Department of Microbiology, College of Medicine, Ewha Womans University, Seoul, 07804, Republic of Korea; [3]Department of Pathology, National Cancer Center Hospital, National Cancer Center, Goyang, 10408, Re-public of Korea; [4]Department of Cancer Biomedical Science, NCC-GCSP, National Cancer Center, Goyang, 10408, Republic of Korea; [5]Department of Orthopaedic Surgery, Center for Sarcoma, National Cancer Center Hospital, National Cancer Center, Goyang, 10408, Republic of Korea; [6]Rare and Pediatric Cancer Branch, Division of Rare and Refractory Cancer, Research Institute, National Cancer Center, Goyang, 10408, Republic of Korea; [7]Medical Engineering Branch, Division of Technology Convergence, Research Institute, NCC-GCSP, National Cancer Center, Goyang 10408, Republic of Korea; [8]Department of Public Health & AI, National Cancer Center-Graduate School of Cancer Science and Policy, National Cancer Center, Goyang 10408, Republic of Korea; [9]Surgical Oncology Branch, Division of Clinical Research, Research Institute, National Cancer Center, Goyang 10408, Republic of Korea; [10]Bioinformatics Branch, Division of Cancer Data Science, Research Institute, National Cancer Center, Goyang 10408, Republic of Korea

*Correspondence: hjyou@ncc.re.kr; Tel.: +82-31-920-2296

†These authors contributed equally to this work.

Supplementary Materials

Four Tables

One Figure

Supplementary Table S1. Differentially Expressed Genes Overlapping with the MSigDB Hallmark EMT Gene Set

Gene	Description	Hallmark EMT genes in US tissues (n=101)	US cell line-enriched genes (n=21)	Shared spatial cluster genes (n=7	Membrane-localized genes (n=2)
CD44	CD44 molecule	O	O	O	O
EMP3	epithelial membrane protein 3	O	O	O	O
COL1A1	collagen type I alpha 1 chain	O	O	O	
COL1A2	collagen type I alpha 2 chain	O	O	O	
FN1	fibronectin 1	O	O	O	
LGALS1	galectin 1	O	O	O	
VIM	vimentin	O	O	O	
BASP1	brain abundant membrane attached signal protein 1	O	O		
COL6A2	collagen type VI alpha 2 chain	O	O		
FLNA	filamin A	O	O		
ITGB1	integrin subunit beta 1	O	O		
MMP2	matrix metallopeptidase 2	O	O		
POSTN	periostin	O	O		
PPIB	peptidylprolyl isomerase B	O	O		
SERPINE1	serpin family E member 1	O	O		
SERPINH1	serpin family H member 1	O	O		
SPARC	secreted protein acidic and cysteine rich	O	O		
TGFBI	transforming growth factor beta induced	O	O		
TIMP1	TIMP metallopeptidase inhibitor 1	O	O		
TNFRSF12A	TNF receptor superfamily member 12A	O	O		
TPM4	tropomyosin 4	O	O		
ADAM12	ADAM metallopeptidase domain 12	O			
ANPEP	alanyl aminopeptidase, membrane	O			
BGN	biglycan	O			
BMP1	bone morphogenetic protein 1	O			
CALD1	caldesmon 1	O			
CALU	calumenin	O			
CAPG	capping actin protein, gelsolin like	O			
CDH11	cadherin 11	O			
CDH2	cadherin 2	O			
COL11A1	collagen type XI alpha 1 chain	O			
COL12A1	collagen type XII alpha 1 chain	O			
COL16A1	collagen type XVI alpha 1 chain	O			
COL3A1	collagen type III alpha 1 chain	O			
COL4A1	collagen type IV alpha 1 chain	O			
COL4A2	collagen type IV alpha 2 chain	O			
COL5A1	collagen type V alpha 1 chain	O			
COL5A2	collagen type V alpha 2 chain	O			
COL5A3	collagen type V alpha 3 chain	O			
COL6A3	collagen type VI alpha 3 chain	O			
COLGALT1	collagen beta(1-O)galactosyltransferase 1	O			
COPA	COPI coat complex subunit alpha	O			
CTHRC1	collagen triple helix repeat containing 1	O			
DAB2	DAB adaptor protein 2	O			
ECM1	extracellular matrix protein 1	O			
EDIL3	EGF like repeats and discoidin domains 3	O			
ENO2	enolase 2	O			
FAP	fibroblast activation protein alpha	O			
FAS	Fas cell surface death receptor	O			
FBLN1	fibulin 1	O			
FBN1	fibrillin 1	O			
FBN2	fibrillin 2	O			
FOXC2	forkhead box C2	O			
FSTL1	follistatin like 1	O			
GEM	GTP binding protein overexpressed in skeletal muscle	O			
GJA1	gap junction protein alpha 1	O			
GLIPR1	GLI pathogenesis related 1	O			
HTRA1	HtrA serine peptidase 1	O			
IGFBP2	insulin like growth factor binding protein 2	O			
IGFBP3	insulin like growth factor binding protein 3	O			
IGFBP4	insulin like growth factor binding protein 4	O			
ITGA2	integrin subunit alpha 2	O			
ITGA5	integrin subunit alpha 5	O			
LAMA1	laminin subunit alpha 1	O			
LOX	lysyl oxidase	O			
LOXL1	lysyl oxidase like 1	O			
LOXL2	lysyl oxidase like 2	O			
LRP1	LDL receptor related protein 1	O			
LRRC15	leucine rich repeat containing 15	O			
MCM7	minichromosome maintenance complex component 7	O			
MEST	mesoderm specific transcript	O			
MMP14	matrix metallopeptidase 14	O			
MXRA5	matrix remodeling associated 5	O			
MYLK	myosin light chain kinase	O			
NID2	nidogen 2	O			
NNMT	nicotinamide N-methyltransferase	O			
NOTCH2	notch receptor 2	O			
NT5E	5'-nucleotidase ecto	O			
NTM	neurotrimin	O			
PCOLCE	procollagen C-endopeptidase enhancer	O			
PDLIM4	PDZ and LIM domain 4	O			
PLAUR	plasminogen activator, urokinase receptor	O			
PLOD1	procollagen-lysine,2-oxoglutarate 5-dioxygenase 1	O			
PLOD2	procollagen-lysine,2-oxoglutarate 5-dioxygenase 2	O			
PLOD3	procollagen-lysine,2-oxoglutarate 5-dioxygenase 3	O			
RGS4	regulator of G protein signaling 4	O			
SAT1	spermidine/spermine N1-acetyltransferase 1	O			
SCG2	secretogranin II	O			
SDC1	syndecan 1	O			
SERPINE2	serpin family E member 2	O			
SLIT2	slit guidance ligand 2	O			
SNAI2	snail family transcriptional repressor 2	O			
SPP1	secreted phosphoprotein 1	O			
TGFB1	transforming growth factor beta 1	O			
THBS1	thrombospondin 1	O			
THBS2	thrombospondin 2	O			
THY1	Thy-1 cell surface antigen	O			
TNC	tenascin C	O			
VCAM1	vascular cell adhesion molecule 1	O			
VCAN	versican	O			
WIPF1	WAS/WASL interacting protein family member 1	O			

Supplementary Table S2. Cox Regression Analysis of Clinical Factors and EMP3 RNA Expression for Survival in the TCGA Pan-Cancer Atlas (Sarcoma, UPS Subtype).

	OS							DFS						
		Univariable Analysis			Multivariable Analysis				Univariable Analysis			Multivariable Analysis		
	n (%)	HR	95% CI	*p*-value	HR	95% CI	*p*-value	*n* (%)	HR	95% CI	*p*-value	HR	95% CI	*p*-value
Total	49 (100)							27 (100)						
EMP3		0.76	0.45-1.28	0.297	0.84	0.47-1.52	0.565		0.59	0.26-1.31	0.194	0.59	0.26-1.36	0.217
Diagnosis Age		1.05	1.00-1.10	0.058	1.05	1.00-1.11	0.038		1.02	0.96-1.08	0.563	1.02	0.96-1.10	0.506
ICD10_classification														
C49.2	26 (53)	0.68	0.24-1.90	0.463	1.26	0.39-4.12	0.697	14 (52)	0.98	0.26-3.68	0.977	1.95	0.43-8.84	0.387
Others	23 (47)	ref			ref			13 (48)	ref			ref		
Sex														
Male	24 (49)	1.95	0.69-5.47	0.205	2.6	0.79-8.47	0.114	11 (41)	2.51	0.67-9.44	0.175	3.63	0.78-16.98	0.101
Female	25 (51)	ref			ref				16 (59)	ref		16 (59)	ref	
	DSS							PFS						
		Univariable Analysis			Multivariable Analysis				Univariable Analysis			Multivariable Analysis		
	n (%)	HR	95% CI	*p*-value	HR	95% CI	*p*-value	*n* (%)	HR	95% CI	*p*-value	HR	95% CI	*p*-value
Total	49 (100)							49 (100)						
EMP3		0.67	0.37-1.21	0.183	0.71	0.34-1.49	0.369		0.69	0.46-1.02	0.064	0.61	0.39-0.98	0.040
Diagnosis Age		1.05	0.99-1.11	0.085	1.06	1.00-1.12	0.055		1	0.96-1.04	0.914	1	0.96-1.04	0.985
ICD10_classification														
C49.2	26 (53)	0.68	0.21-2.28	0.537	1.58	0.38-6.51	0.527	26 (53)	1.29	0.55-3.03	0.56	2.09	0.82-5.29	0.121
Others	23 (47)	ref			ref			23 (47)	ref			ref		
Sex														
Male	24 (49)	2.2	0.66-7.35	0.201	3.13	0.78-12.53	0.106	24 (49)	2.03	0.86-4.76	0.105	2.6	1.05-6.42	0.039
Female	25 (51)	ref			ref			25 (51)	ref			ref		

OS, overall survival; DFS, disease-free survival; DSS, disease-specific survival; PFS, progression-free survival; HR, hazard ratio; CI, confidence interval.

Method:

Data were obtained from cBioPortal. Univariable and multivariable Cox proportional hazards models were used to assess associations between clinical variables (sex, ICD-10 classification, diagnostic age, and EMP3 RNA expression) and survival outcomes (OS, DFS, DSS, and PFS). Statistical analyses were performed using R (v4.4.1) with the survival package. All tests were two-sided, and p-values < 0.05 were considered statistically significant.

Supplementary Table S3. Primers for RT-PCR or qRT-PCR

Gene	Direction	Species	MW	Tm	Sequence(5'>3')	bp	NM.	Anealing T (℃)
N-cardherin	Forward	Human	6182	59.3	GGACAGTTCCTGAGGGATCA	253	NM_001792.4	56
	Reverse		6090	57.3	GGATTGCCTTCCATGTCTGT			
TJP1-1	Forward	Human	6235	59.3	GGAGAGGTGTTCCGTGTTGT	253	NM_003257.4	56
	Reverse		6102	59.3	GAGCGGACAAATCCTCTCTG			
Vimentin	Forward	Human	7374	59.3	TGTCCAAATCGATGTGGATGTTTC	117	NM_003380.4	56
	Reverse		6908	60.6	TTGTACCATTCTTCTGCCTCCTG			
ZEB1	Forward	Human	6206	57.3	TGCACTGAGTGTGGAAAAGC	237	NM_001323650.1	53
	Reverse		6246	57.3	TGGTGATGCTGAAAGAGACG			
Slug	Forward	Human	7880	60.1	TGCAATAAGACCTATTCTACGTTCTC	119	NM_003068.4	53
	Reverse		5988	57.3	CCCAGGCTCACATATTCCTT			
Snai1	Forward	Human	6031	59.3	CCCCAATCGGAAGCCTAACT	158	NM_005985.3	58
	Reverse		6176	61.4	GGACAGAGTCCCAGATGAGC			
Twist1	Forward	Human	6158	61.4	GGAGTCCGCAGTCTTACGAG	162	NM_000474.3	58
	Reverse		6133	59.3	CCAGCTTGAGGGTCTGAATC			
EMP3	Forward	Human	6020	59	GTCTCTTCTAGCCACCGGC	92	NM_001425.3	58
	Reverse		6149	61	GATCTCCTCGGCGTGAATGG			
GAPDH	Forward	Human	6056	58.1	CTGACCTGCCGTCTAGAAAA	225	NM_002046.7	55
	Reverse		6106	57.5	GCCAAATTCGTTGTCATACC			

Supplementary Table S4. Antibodies for immunoblotting or Immunohistochemistry

Target (Protein)	Company (Catalog No.)	Host	MW (kDa)	Dilution (WB)
α-SMA	Abcam (ab32579)	Mouse	~42	1:1,000
AKT	CST (9277s)	Rabbit	~60	1:1,000
BACH1	Abcam (ab33059)	Rabbit	~90	1:1,000
β-Actin	Santa Cruz (sc-69879)	Mouse	~42	1:5,000
E-Cadherin	CST (3195s)	Rabbit	~120	1:1,000
EMP3	Abcam (ab236671)	Rabbit	~18	1:1,000
ERK1/2	CST (4695S)	Rabbit	42/44	1:1,000
N-Cadherin	CST (4061S)	Rabbit	~88–100	1:1,000
p-AKT (Ser473)	CST (9271s)	Rabbit	~60	1:1,000
p-AKT (Thr308)	CST (9275s)	Rabbit	~60	1:1,000
p-ERK1/2	CST (4370S)	Rabbit	42/44	1:1,000
PDGFRα	Abcam (ab71009)	Rabbit	~170	1:1,000
PDGFRβ	Santa Cruz (sc-374573)	Mouse	~180–190	1:1,000
p-SMAD2	CST (3108S)	Rabbit	~58	1:1,000
SMAD2	CST (5339)	Rabbit	~58	1:1,000
SNAI1	CST (3879S)	Rabbit	~29	1:1,000
TWIST1	Santa Cruz (sc-15393)	Mouse	~25	1:1,000
Vimentin	Santa Cruz (sc-6260)	Mouse	~54	1:1,000
ZO-1 (TJP1)	Invitrogen (617300)	Mouse	~220	1:1,000

Supplementary Figures and Legends

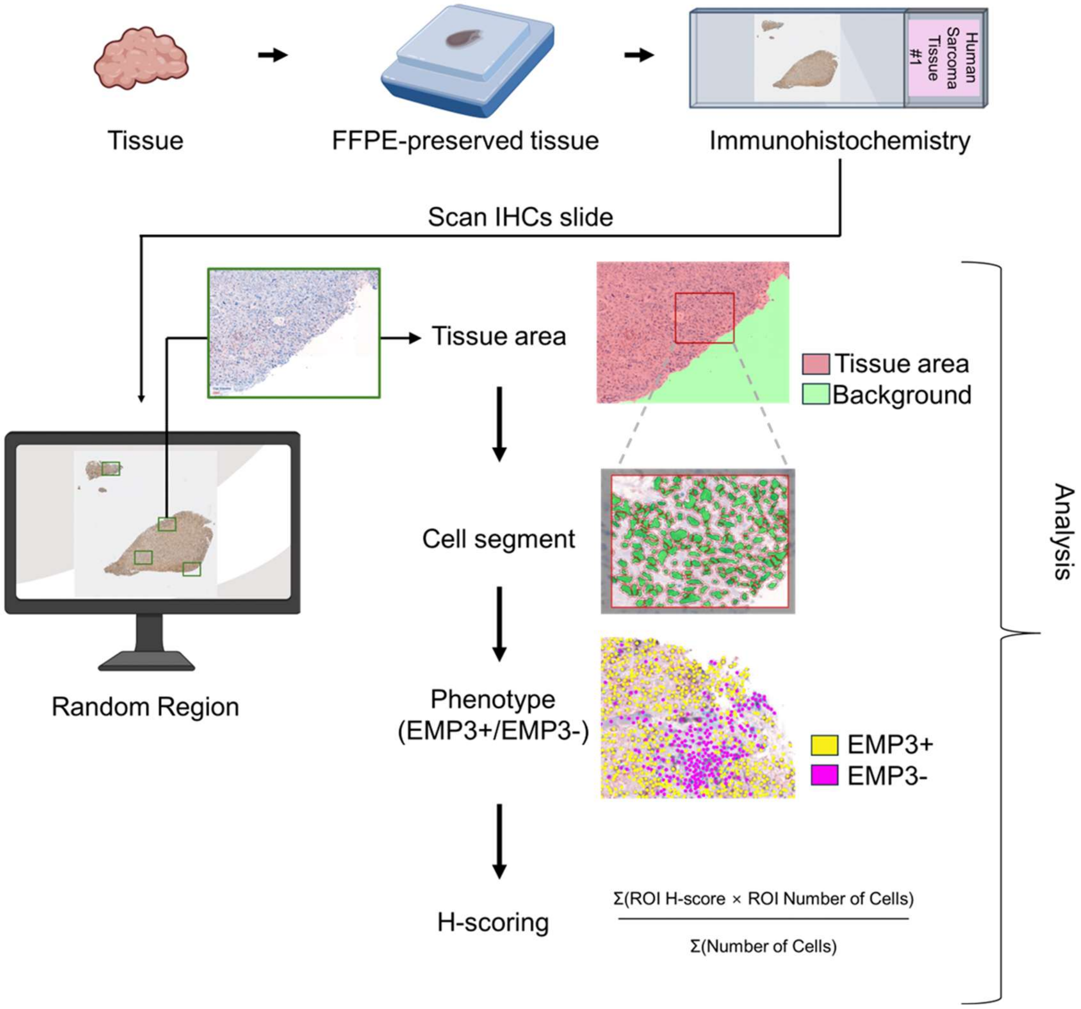

Supplementary Figure S1. Schematic overview of InForm analysis and H-score quantification for immunohistochemical evaluation. Workflow illustrating the digital image analysis pipeline using InForm software for quantitative assessment of immunohistochemistry (IHC) staining and calculation of H-scores. The figure was created with BioRender.com.
